# In Vitro and In Vivo Digestibility of Soybean, Fish, and Microalgal Oils, and Their Influences on Fatty Acid Distribution in Tissue Lipid of Mice

**DOI:** 10.3390/molecules25225357

**Published:** 2020-11-17

**Authors:** Bo-Ram Na, Jeung-Hee Lee

**Affiliations:** Department of Food and Nutrition, Daegu University, Gyeongsan-si 38453, Korea; skqhfka1020@naver.com

**Keywords:** microalgal oil, DHA, in vitro digestion, apparent digestibility

## Abstract

The digestion rates of microalgal (docosahexaenoic acid, DHA, 56.8%; palmitic acid, 22.4%), fish (DHA, 10.8%; eicosapentaenoic acid, EPA, 16.2%), and soybean oils (oleic, 21.7%; linoleic acid, 54.6%) were compared by coupling the in vitro multi-step and in vivo apparent digestion models using mice. The in vitro digestion rate estimated based on the released free fatty acids content was remarkably higher in soybean and fish oils than in microalgal oil in 30 min; however, microalgal and fish oils had similar digestion rates at longer digestion. The in vivo digestibility of microalgal oil (91.49%) was lower than those of soybean (96.50%) and fish oils (96.99%). Among the constituent fatty acids of the diet oils, docosapentaenoic acid (DPA) exhibited the highest digestibility, followed by EPA, DHA, palmitoleic, oleic, palmitic, and stearic acid, demonstrating increased digestibility with reduced chain length and increased unsaturation degree of fatty acid. The diet oils affected the deposition of fatty acids in mouse tissues, and DHA concentrations were high in epididymal fat, liver, and brain of mice fed microalgal oil. In the present study, microalgal oil showed lower in vitro and in vivo digestibility, despite adequate DHA incorporation into major mouse organs, such as the brain and liver.

## 1. Introduction

DHA (C22:6) is a major n-3 polyunsaturated fatty acid (PUFA) that is uniquely concentrated in the brain, nervous tissues, and retina. DHA is essential for normal neurological development and function [1]. It is known to lower the risk of coronary artery disease [2], inhibit the onset of cardiac arrhythmia and cardiovascular disease [3,4], and prevent the accumulation of beta-amyloid that damages brain cells in patients with dementia [5]. Fish oil contains EPA (13.3%) and DHA (8.9%), while microalgal oil extracted from *Schizochytrium* sp. has high DHA (54.9%) and DPA (n-6; 11.9%) contents but low EPA content (0.8%) [6].

After ingestion, dietary oil is emulsified with bile acid and the *sn*-1,3 fatty acids (FAs) of triacylglycerol (TAG) are hydrolyzed by *sn*-1,3 specific pancreatic lipases, and then absorbed by small intestinal mucosal cells in the form of 2-monoacylglycerol (MAG) and free fatty acids (FFAs) [7,8]. Oil is composed of different types of FAs and the position of FAs in the glycerol backbone of TAG varies, leading to differences in digestibility, absorption, and bioavailability after ingestion. Short-chain FAs exhibit higher degrees of hydrolysis by lipase than long-chain FAs. Unsaturated FAs show higher degrees of lipase-mediated hydrolysis than saturated FAs; in particular, PUFA, which has a double bond close to the carboxyl group, has a lower hydrolysis rate [8]. Microalgal oil contains a large amount of DHA and when ingested, it has a low hydrolysis rate. So, the digestibility and absorption rate of ingested microalgal oil is low, resulting in reduced bioavailability of DHA.

The digestibility of oil was evaluated using in vitro and in vivo digestion methods. In vitro digestion of oil was performed using a multi-step digestion model that simulated the complex digestion processes (oral, gastric, and intestinal) of the human body, step by step, to measure the sizes of hydrolyzed fat globules and the content of released FFA or unhydrolyzed TAG [9,10]. Recent studies that applied in vitro multi-step digestion models include a comparison of digestibility between symmetric TAG and asymmetric TAG [11], analysis of digestibility among various real foods (dairy, meat, fish, egg, nuts, oils, fats, etc.) [12], and evaluation of the impact of emulsifier type on lipid hydrolysis of soybean oil-in-water emulsions [13].

In vivo digestion of dietary oil was assessed using the apparent digestibility coefficient obtained from animal studies, which is a measure of bioavailability expressed as the percentage of ingested oil that was not excreted in feces; in this method, chromic oxide was used as an indicator of the indigestible marker [14]. Kaplan and Greenwood [15] reported that the digestibility coefficient of dietary lipids in rats was the highest for medium-chain triglyceride oil (98.7%), followed by soybean oil (97.0%), hydrogenated coconut oil (94.5%), and fully hydrogenated soybean oil (30.9%). Digestibility is affected by the fatty acid profile; in particular, the low digestibility of hydrogenated oil could be attributed to the poor hydrolysis of stearic acid. Sugano and Imaizumi [16] investigated the effect of the saturation degree on dietary fat digestibility in rats, and reported that lauric acid fat (97.5%) and myristic acid fat (96.4%) had the highest digestibility while stearic acid fat (84.0%) had the least. Previous in vivo lipid digestibility studies were mainly focused on soybean oil, tallow, coconut oil, palm oil, canola oil, and liver cod oil fed to rats, fishes, pigs, and chickens [17,18,19]; however, studies on microalgal oil with high DHA content are limited.

In vitro and in vivo digestion of oils containing DHA and its tissue disposition after ingestion have been studied. The in vitro digestion rate of microalgal oil (TAG form) is lower than that of soybean oil but higher than that of DAG rich microalgal oil, and oil-in-water emulsion increases in vitro digestibility [20]. Tou et al. [21] reported that krill oil with high EPA and DHA contents had lower apparent digestibility than salmon, tuna, and menhaden oils. In mice fed oil containing 5% DHA for 1 week, DHA concentrations in the brain and liver were higher than those in the control group [22]. DHA contents in the livers and brains of rats fed oil containing 1.3% DHA for 8 weeks were significantly higher than those of the control diet group [23]. The DHA content in the brains of rats fed with krill oil was similar to that of the group fed corn oil and menhaden oil but significantly lower than that of the group fed salmon oil and tuna oil; the DHA content in the liver of the krill oil-fed group was significantly higher than that of the corn oil-fed group [21].

Owing to the numerous health benefits of DHA, the consumption of oils containing it is increasing and thus, the need to supply oils containing high concentrations of DHA is emerging. However, the DHA source and content, and the period of intake affect digestibility, absorption, and tissue deposition, resulting in different bioavailability. Therefore, research on the digestibility rates of oils containing high DHA concentrations and their efficient incorporation into tissues is necessary. In this study, the digestion rates of microalgal oil containing high DHA content and fish oil containing low DHA and EPA contents were compared by coupling in vitro multi-step digestion and in vivo apparent digestion models using mice. In addition, we investigated whether DHA was efficiently deposited in each tissue by analyzing the FA compositions of the brain, liver, and epididymal fat after ingestion of the oils.

## 2. Results and Discussion

### 2.1. Fatty Acids Composition of the Oils

The major FAs in soybean oil are linoleic (C18:2, 54.6%), oleic (C18:1, 21.7%), palmitic (C16:0, 10.9%), linolenic (C18.3, 6.6%), and stearic acid (C18:0, 4.5%), and the main FAs at the *sn*-2 position are linoleic (46.9%), oleic (22.0%), and linolenic acid (5.8%) (Table 1). The major FAs in fish oil are palmitic (18.5%), EPA (16.2%), palmitoleic (C16:1, 13.1%), DHA (10.8%), myristic (9.2%), and oleic acid (8.8%), showing that this oil is rich in the *n*-3 FAs of EPA and DHA; the main FAs at the *sn*-2 position include palmitic (20.0%), EPA (17.3%), palmitoleic (12.3%), DHA (19.0%), and myristic acid (9.8%). Microalgal oil mainly contains DHA (56.8%), DPA (12.3%), and palmitic (22.4%), whereas EPA is present in trace amounts (0.6%); at the *sn*-2 position, it contains DHA (67.3%), DPA (15.2%), and palmitic acid (11.7%). The DHA and DPA contents were 5.3. and 4.7 times higher in microalgal oil than in fish oil, respectively, whereas EPA was 25.7 times higher in fish oil. The fish oil used in the present study was obtained from menhaden, and the fatty acid composition was similar to commercial Atlantic and Gulf coast menhaden oils [24] (Joseph, 1985). The microalgal oil is a commercial water-extracted Chromista algae oil (*Schizochytrium* sp.).

Generally, long-chain unsaturated fatty acids (LC-USFAs) tend to be located at the *sn*-2 position and saturated fatty acids (SFAs) with a relatively small number of carbons are located at the *sn*-1,3 position of the TAG structure. In soybean oil, C18-USFAs and C16- and C18-SFAs respectively account for 77.4% and 22.5% of the FAs at *sn*-1,3 and 98.5% and 1.4% at *sn*-2. Fish oil and microalgal oil are composed of FAs with various carbon numbers and double bonds. At *sn*-2 and *sn*-1,3, PUFAs with C20 or higher account for 47.8% and 31.8% of FAs, respectively, in fish oil and 83.5% and 65.8%, respectively, in microalgal oil. Therefore, the constituent FAs in each oil are different with different locations in the glycerol backbone, which affects digestion and absorption upon ingestion.

### 2.2. In Vitro Digestion of the Oils

**Figure 1:**
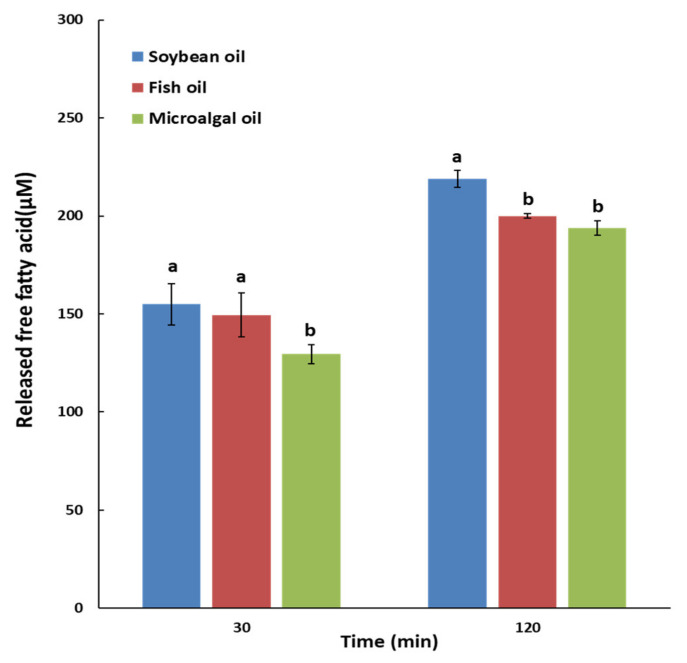
Released free fatty acids (µM) of soybean oil, fish oil, and microalgal oil in an in vitro multi-step digestion model. ^a,b^ Different letters above the bars at the same digestion time indicate significant differences by Duncan’s multiple range test at *p* < 0.05.

The degree of oil hydrolysis was expressed in terms of the released free fatty acid (FFA) content (µM) via a step-by-step in vitro simulation of the digestion process with saliva, gastric, duodenal, and bile juices, lasting 30 and 120 min (Figure 1). The released FFA content at 30 min was significantly higher in soybean oil and fish oil than in microalgal oil, and that at 120 min was significantly higher in soybean oil than in fish oil and microalgal oil (*p* < 0.05).

In the body, the digestibility and absorption rates of oils differ due to variations in FA unsaturation, carbon length, and location in the glycerol backbone (*sn*-1,3 or *sn*-2). These variations affect the bioavailability of FAs and lipid metabolism [8]. Oil is emulsified by bile acid after ingestion and absorbed after being hydrolyzed by pancreatic lipase. Most FAs at *sn*-1,3 of TAG are hydrolyzed during digestion, whereas 22% of FAs at the *sn*-2 position are hydrolyzed, which is due to the regiospecificity of pancreatic lipase [7]. The degree of USFA hydrolysis by lipase is greater than that of SFA. Moreover, the degree of lipase-mediated short-chain (SC) FA hydrolysis is higher than that of LCFA. Notably, the hydrolysis rate of PUFA is reportedly low when the double bond is closer to the carboxyl group [8].

In the present study, the PUFA content at *sn*-1,3 was 1.9 times and 1.2 times higher in microalgal oil than in fish oil and soybean oil, respectively; in particular, microalgal oil has 2.0 times more PUFA with carbon length ≥ 20 than fish oil, while soybean oil has none. The double bond present in the FA structure and the carboxyl group were closest in DHA (C22:6, Δ4,7,10,13,16,19) and DPA (C22:5, Δ4,7,10,13,16), followed by EPA (C20:5, Δ5,8,11,14,17), linoleic (C18:2, Δ9,12), and linolenic acid (C18:3, Δ9,12,15). Bottino et al. [25] reported that DHA exhibited greater resistance to pancreatic lipase than EPA, and this phenomenon can be explained by the structural differences between these molecules. In other words, if the terminal methyl and carboxyl groups of PUFA are close, there is a steric hindrance effect on the lipase-mediated hydrolysis. Therefore, the higher polyunsaturation of FAs located in *sn*-1,3 and the double bond close to the carboxyl group inhibit the TAG hydrolysis activity of lipase, resulting in lower digestion rates of microalgal oil and fish oil compared to that of soybean oil. Aarak et al. [26] analyzed the profile of released FAs after in vitro digestion of salmon oil with human duodenal juice and a commercial enzyme preparation (porcine pancreatin and bile), and reported that the concentration of linoleic acid was the highest, followed by EPA and DHA (*p* < 0.05).

In an in vitro hydrolysis study by Ikeda et al. [27], wherein tridocosahexaenoyl glycerol (TriDHA) and trieicosapentaenoyl glycerol (TriEPA) were treated with porcine pancreatic lipase, the hydrolysis rates of TriEPA and TriDHA in the initial period were low, but it increased markedly afterwards; in the end, most TriEPA and 80% of TriDHA were hydrolyzed. Similarly, in this study, when the duration of in vitro digestion was increased from 30 to 120 min, the released FFA contents of soybean oil and fish oil increased by 1.41 and 1.34 times, respectively, whereas that of microalgal oil (51.5% DHA at *sn*-1,3; 67.3% at *sn*-2) increased by 1.53 times, indicating that longer digestion led to higher hydrolysis of DHA present in TAG at high concentrations.

### 2.3. Mice Body Weights, Tissue Weight, and Crude Lipid Content in Tissue

The body weights of mice increased in all four groups during the 4-week period. The body weight gain (3.28~5.55 g) and feed efficiency ratio (FER, 4.23~6.54%) of mice fed the experimental diet were significantly higher than that of the normal group (0.37 g, 0.48%) (*p* < 0.05), and there was no significant difference in body weight gain and FER among the SO, FO, and MO groups (*p* > 0.05) (Table 2). Experimental diet-fed mice had significantly heavier liver and epididymal fat than normal diet-fed mice; the liver weight was the highest in the MO group and lowest in the SO group (*p* < 0.05). There were no significant differences in brain and heart weights among the diet groups (*p* > 0.05) (Table 2). The crude lipid contents of the brain and epididymal fat were similar in all four diet groups (*p* > 0.05), while the lipid content of the liver was significantly lower in the FO compared to those in the SO and MO groups (*p* < 0.05) (Table 2). According to the study of Yuan et al. [28], the rat supplemented with 10% fish oil in a high-fat high-cholesterol diet showed a significantly reduced hepatic triacylglycerol content than the 10% lard diet, demonstrating that fish oil protects against high-fat high-cholesterol diet-induced non-alcoholic fatty liver disease by improving lipid metabolism and ameliorating hepatic inflammation.

### 2.4. Apparent Digestibility of the Oils and Individual Fatty Acids

The apparent digestibility values were determined by measuring the coefficients of digestibility for soybean oil, fish oil, and microalgal oil in mice (Table 3). The fecal crude lipid content in each group was in the following order: microalgal oil (15.87%) > fish oil (9.24%), soybean oil 20% (8.89%) > soybean oil 7% (6.87%). The fecal lipid content in the microalgal oil intake group was significantly higher than in the other groups (*p* < 0.05). The apparent digestibility of the soybean oil 20% (SO) was significantly higher than that of the 7% intake group (NO) (*p* < 0.05). Among the experimental diet groups, the apparent digestibility of the microalgal oil group (91.49%) was significantly lower than the others (*p* < 0.05), and there was no significant difference between the soybean oil (96.50%) and fish oil (96.99%) groups (*p* > 0.05). This suggests that microalgal oil, due to its lower digestibility and absorption rate compared to soybean oil and fish oil, was not be absorbed easily in the body and is secreted as feces, resulting in low apparent digestibility.; This is consistent with the results of the in vitro digestibility model (Figure 1). According to the in vivo study of Tou et al. [21] using rats, krill oil (93.1%) had the highest EPA and DHA contents but lower apparent digestibility compared to salmon oil (98.8%), tuna oil (98.0%), and menhaden oil (97.2%). The EPA and DHA concentrations in krill oil were 19.92~22.83% and 10.60~12.33%, respectively [29], and its DHA concentration was lower than that of microalgal oil in the present study (56.8%).

Table 3 shows the apparent digestibility values of individual FAs in each diet group. In the soybean oil 7% and soybean oil 20% groups, the digestibility coefficient of individual FA was significantly higher for C18:3 (96.2~96.9%), followed by C18:2 (93.6~94.7%), C18:1 (91.5~92.7%), C16:0 (88.2~88.4%), and C18:0 (85.9~86.5%) (*p* < 0.05). FA digestibility in the fish oil group was significantly higher for C20:5 (95.7%), followed by C22:6 (94.5%), C16:1 (93.8%), C18:1 (92.7%), C16:0 (89.2%), and C18:0 (76.8%) (*p* < 0.05). FA digestibility in the microalgal oil group was in the following order: C22:5 (99.9%) > C22:6 (93.2%) > C16:0 (80.8%) (*p* < 0.05). Generally, FA digestibility increased with reduced chain length and further increased with increasing unsaturation level [8]. In all groups, the digestibility coefficients of palmitic acid and stearic acid were significantly lower than those of other unsaturated FAs, because the long-chain SFA positioned at *sn*-1,3 in each oil has a lower rate of pancreatic lipase-mediated hydrolysis; during digestion, free palmitic and stearic acids form insoluble calcium soap and are excreted in feces [8]. In fish oil, EPA and DHA had higher digestibility than oleic acid and palmitoleic acid, and this was in agreement with the results reported by Ikeda et al. [27], indicating that n-3 FAs are slowly released from the *sn*-1,3 positions by pancreatic lipase at the initial period of digestion, but as digestion progresses, the hydrolysis rate increases significantly and may be even higher than that of oleic acid. In the microalgal oil group, the digestibility of DPA was significantly higher than that of DHA, since DHA at the *sn*-1,3 position resists pancreatic lipase hydrolysis, whereas DPA is hydrolyzed more easily [25].

### 2.5. Fatty Acids Distribution in Feces and Tissues

#### 2.5.1. Fatty Acid Profile in Fecal Lipid

The distribution of fecal FAs in mice fed soybean oil diets was substantially different from those in mice fed fish oil or microalgal oil diets (Table 4). The fecal FA profile in the SO group consisted of linoleic (38.8%), oleic (20.5%), palmitic (14.1%), stearic (7.1%), and linolenic acid (2.8%). In the microalgal oil group, DHA (36.4%), palmitic (40.2%), and stearic acid (5.5%) constituted the fecal FAs profile, while palmitic acid (24.3%), DHA(7.3%), EPA(8.6%), palmitoleic (9.9%), stearic (9.1%), and oleic acid (7.8%) were included in the fecal FAs profile of the fish oil group. DPA, EPA, DHA, palmitoleic, linoleic, oleic, and linolenic acids with high apparent digestibility seem to be well absorbed in the body due to effective hydrolysis by pancreatic lipase, and the amount of these FAs excreted via feces was lower; therefore, these FA compositions of fecal lipid were relatively lower than those of diet oils. On the other hand, palmitic and stearic acids have lower hydrolysis rates and form insoluble soap with calcium; thus, these FAs were not easily absorbed, leading to excretion via feces, and so their composition profile in fecal lipids was higher than that of diet oil. Therefore, FAs with high digestibility in the diet are released well as FFA, and their absorption rate is high, which leads to a decrease in the amount excreted in feces, resulting in a low composition in the fecal FA distribution. On the contrary, FAs with low digestibility cannot be well-hydrolyzed and are excreted more, which leads to a higher composition of fecal lipids.

#### 2.5.2. Fatty Acid Profile in Epididymal Fat

The FA composition of epididymal fats in the NO and SO groups showed that the predominant FAs were palmitic (13.1~19.2%), oleic (31.2~34.2%), linoleic acid (32.2~43.9%), and small amounts of C16:1 (2.1~4.3%) and stearic acid (1.9~2.4%) (Table 4). The major FAs distributed in the fish oil group were palmitic (29.5%), oleic (21.8%), linoleic (10.9%), and palmitoleic acids (10.5%), and small amounts of stearic (3.8%), EPA (2.3%), and DHA (2.8%). The major fatty acids in the microalgal oil group were palmitic (36.2%), oleic (17.4%), linoleic (12.7%), and DHA (17.1%), and small amounts of C16:1 (3.4%), DPA (2.9%), and stearic acid (3.0%). The depot fats of animals generally consist of palmitic and oleic acids, with varying amounts of myristic, palmitoleic, stearic, and linoleic acids [30]. In the present study, palmitic and oleic acids were mainly distributed in the epididymal fats of all four groups, and specific FAs, such as linolenic acid in the soybean oil 20% and soybean oil 7% groups; palmitoleic acid, EPA, and DHA in the fish oil group; and DPA and DHA in the microalgal oil group. Since depot fats originate from both endogenously synthesized and exogenous FAs in the blood stream, the FA composition of adipose tissue is markedly influenced by the dietary ingested oils.

#### 2.5.3. Fatty Acid Profile in Liver Lipid

The liver FAs mainly consisted of palmitic (19.1~26.9%), stearic (8.7~12.8%), oleic (4.7~16.2%), linoleic acid (4.5~33.2%), ARA (6.72~14.4%), and DHA (5.7~40.3%) in the four diet groups (Table 5). PUFAs, such as ARA (n-6), EPA (n-3), DPA (n-6), and DHA (n-3), were generated by a series of elongation and desaturation of linoleic acid (n-6) and linolenic acid (n-3), and distributed in the mouse liver. In addition, the specific fatty acids originating from soybean, fish, and microalgal oils were highly distributed in the liver, such as linoleic acid in the SO group, EPA and DHA in the FO group, and DPA and DHA in the MO group. This result suggested that dietary FAs affected the FA profile of mouse liver lipids. In particular, in the microalgal oil group, higher concentrations of DHA (40.3%) were distributed in the liver, which might be because DHA was incorporated at a considerable concentration (67.3%) at the *sn*-2 position in microalgal oil, and the DHA was maintained in the *sn*-2 position during absorption, intestinal TAG resynthesis, and degradation by lipoprotein lipase, and then transferred to the liver. According to the studies on tissue deposition after ingestion of DHA-containing oil, the DHA concentration in the livers of mice that consumed oil containing 5% DHA for one week was higher than that of the control group, but the difference was not significant [22], and the DHA content in the livers of rats that consumed oil containing 1.3% DHA for 8 weeks was significantly higher than that of the control group [23].

The liver plays a key role in lipid metabolism for FA synthesis and lipid circulation through lipoprotein synthesis. The FAs in the liver originate from different sources: (1) de novo lipogenesis (FA synthesis from excess glucose); (2) lipolysis (hydrolysis of TAG into FAs); (3) uptake of dietary FAs produced by hydrolysis of esterified triacylglycerols, which are transported from gut-derived chylomicron remnants; and (4) uptake of free FA released by lipolysis of adipose tissue [31]. Therefore, the FAs in the liver are derived from various sources, and the FA profile in the mouse liver varied compared to that in mice feces and epididymal fat (Table 4).

#### 2.5.4. Fatty Acid Profile in Brain Lipid

The mouse brain had a higher lipid content than the liver, and the crude lipid contents of mice brains were similar among the four groups (*p* < 0.05) (Table 3). The major FAs distributed in the brain were palmitic (22.1~22.4%), stearic (20.5~21.5%), oleic (16.6~19.6%), ARA (7.8~10.3%), and DHA (15.4~20.4%), and small amounts of linoleic acid (0.3~1.7%), DPA (0.3~1.8%), and EPA (0~0.36%) (Table 5).

The brain has a higher lipid content than other organs, except for adipose tissue, and lipids are mostly present as phospholipids in cell membranes. The brain lipid consists mainly of palmitic, stearic, oleic acid, and PUFA [22], and phospholipids generally comprise PUFAs, such as DHA and ARA, and smaller amounts of EPA and linolenic acid [32]. The brain is capable of synthesizing FAs as efficiently as the liver via either a de novo or a chain elongation-desaturation process of the required FAs, decreasing the demand for transportation of FAs synthesized or ingested in the diet [33]. The most abundant PUFAs in the brain are ARA and DHA, which can be taken up from a circulating lipid pool through the blood-brain barrier (BBB), and synthesized from dietary sources of C18:2 and C18:3, which pass from the blood into the brain through the BBB [33]. EPA and DHA are reported to enter into the brain by crossing the BBB at similar rate [34], but EPA was maintained at a very low level in the brain by multiple pathways of rapid β-oxidation, decreased incorporation, and elongation and desaturation to DPA (n-3) [35].

In the present study, higher concentrations of saturated FAs (palmitic and stearic acids) and MUFA (oleic acid) in the mouse brain appeared to be endogenous rather than diet originated. In mice fed 20% oil diets, microalgal oil significantly increased the DHA and DPA concentrations by 1.3~1.2 and 5.4 times, respectively, compared to soybean oil and fish oil (*p* < 0.05), while soybean oil significantly increased the concentration of linoleic and ARA by 4.3~5.9 and 1.3~2.6 times, respectively, compared to microalgal oil and fish oil. EPA was found in trace amounts only in the fish oil and microalgal oil diet groups. Similar to the results of this study, a significant increase in the brain DHA level was observed in dietary DHA oil-fed rats compared to control-fed rats [36]. DHA was also incorporated in the brains of rats that were fed corn oil and flaxseed oil that did not contain DHA, although the level was significantly lower than that of rats fed salmon oil and tuna oil, which contained DHA [21]. Thus, dietary FAs could influence the composition of mouse brain lipids.

## 3. Materials and Methods

### 3.1. Materials

Microalgal oil extracted from *Schizochytrium* sp. was purchased from Source-Omega LLC. (Chapel Hill, NC, USA). Fish oil extracted from menhaden was purchased from Sigma-Aldrich Chemical Co. (St. Louis, MO, USA), and soybean oil was purchased from Ottogi (Pyeongtaek, Gyeonggi-do, Korea). Bile salts, α-amylase, lipase from porcine pancreas, bovine serum albumin, pancreatin from porcine pancreas, mucin from porcine stomach, sodium molybdate, chromic oxide, and potassium dichromate were purchased from Sigma-Aldrich Chemical Co. Triundecanoin, a standard for fatty acid analysis, was purchased from NU-CHEK PREP, Inc. (Elysian, MN, USA) and Supelco 37 Component FAME Mix was purchased from Supelco Inc. (Bellefonte, PA, USA).

### 3.2. Analysis of Fatty Acid Composition

Samples (50 mg), 1 mL triundecanoin in isooctane (5 mg/mL), and 1.5 mL of 0.5 N methanolic NaOH were added to a test tube, followed by saponification at 85 °C for 10 min. After cooling, 2 mL of BF_3_-methanol were added, then methylated at 85 °C for 10 min and cooled. Then, 2 mL of isooctane were added and vortexed for 1 min, and 1 mL of saturated NaCl solution was added. After centrifugation (1224× *g*, 5 min), the supernatant was passed through a sodium sulfate column to remove moisture. The FA composition was analyzed using gas chromatography equipped with a flame ionization detector (GC-2010 Plus, Shimadzu Corp., Kyoto, Japan) and SP^TM^-2560 column (100 m × 0.25 mm × 0.2 µm, Supelco Inc.). The oven temperature was maintained at 100 °C for 5 min, increased to 240 °C at 4 °C/min, and maintained for 40 min. The column flow (carrier gas) was 1.00 mL/min (N_2_), the injector and detector temperatures were 250 and 260 °C, respectively, the split ratio was 100:1, and the injection volume was 1 µL. Each FA was identified based on the retention time of the standard chromatogram, and the area of each peak was expressed as % of total FAs.

### 3.3. Analysis of Positional Fatty Acid Composition

Oil (25 mg), tris-HCl buffer (pH 7.6, 25 mL), 0.05% bile salt (6.25 mL), 2.2% CaCl_2_ (2.5 mL), and pancreatic lipase (25 mg) were mixed in a test tube, and allowed to react for 3 min at 37 °C, with 30-s vortexing repeated 3 times. Then, 6 mL of diethyl ether were added and vortexed for 1 min. After centrifugation (1224× *g*, 5 min), the supernatant was passed through an anhydrous sodium sulfate column, concentrated with N_2_, and loaded onto a thin-layer chromatography (TLC) F_254_ silica plate (20 × 20 cm, Merck, Kenilworth, NJ, USA). After separation with a developing solvent (*n*-hexane: diethyl ether: acetic acid = 50:50:1, *v*/*v*/*v*), the 2-MAG band in the TLC plate was taken and analyzed with GC, and positional FAs were calculated using the following equation:

Fatty acid composition at *sn*-1,3 position (%) = [3 × total fatty acid composition (%)—fatty acid composition at *sn*-2 (%)]/2.

### 3.4. In Vitro Multi-Step Digestion Model

The in vitro digestion rate of the oil was measured by modifying the method described by Versantvoort et al. [37] and Chang et al. [20]. All digestive juices were prepared and used on the day of the experiment. Oil (100 mg) and saliva juice (1.2 mL) were mixed in an Erlenmeyer flask at 37 °C and 80 rpm for 5 min, and gastric juice (2.4 mL) was added and reacted for 2 h. NaHCO_3_ solution (0.4 mL), bile juice (1.2 mL), and duodenal juice (2.4 mL) were added, followed by hydrolysis reaction for 30 and 120 min. For control samples (0 min), duodenal juice without pancreatin and lipase were added. After the reaction, the lipase inhibitor 4-bromophenylboronic acid (100 μL) was added to stop digestion. For lipid extraction of the digested solution, *n*-hexane was added and centrifuged at 1763× *g* for 5 min, and the supernatant was passed through an anhydrous sodium sulfate column (repeated 3 times). Next, 1N HCl (0.5 mL) was mixed with the remaining lower part for 1 min, and 10 mL of *n*-hexane were mixed and centrifuged, and the supernatant was removed, and the moisture removed (repeated 3 times). The supernatants were combined, and after removing *n*-hexane with N_2_, 10 mL of ethanol:*n*-hexane (1:1, *v*/*v*), and 1 mL of 1% phenolphthalein were added, and titrated with 50 mM KOH solution. The in vitro digestion rate of each oil was expressed as released free fatty acids (µM) using the following equation [38]:

(1) $$Released\;free\;fatty\;acids\left(\mu M\right)of\;the\;oils=\frac{volume\;of\;KOH\left(mL\right)\times normality\;of\;KOH\left(mN\right)\times 100}{weight\;of\;the\;oil\left(mg\right)}$$

### 3.5. Animals and Diets

Forty male ICR mice, aged 6 weeks (initial body weight of approximately 30 g), were obtained from Samtako BioKorea (Osan, Korea) and housed in a temperature-controlled environment at 22 ± 3 °C and 50 ± 10% relative humidity. The mice were maintained on a 12-h light/dark cycle and provided with a designated diet (3.5 g) and ad libitum access to water. After one week of acclimation with a standard diet (Samtako BioKorea), mice were randomly assigned to one of four dietary treatment groups, and two mice were housed in one cage for 4 weeks. The NO group was fed a normal diet with 7% soybean oil, and SO, FO, and MO groups received experimental diets containing 20% soybean oil, fish oil, and microalgal oil, respectively. Body weight and food intake were measured twice a week by each cage, in which food intake was calculated by subtracting the amount of remaining food from the amount of provided food by each cage. The composition of the diets, based on the AIN-93G DIET conditions, is presented in Table 6.

At week 4 of the diet intervention, a diet containing 0.5% chromic oxide was provided for 5 consecutive days, and green feces were collected. The mice were fasted for 24 h before sacrifice, and dissection was performed between 10:00 am and 12:00 pm. The liver, brain, and epididymal fat of the mice were removed, rinsed, weighed, and stored in a deep freezer (Nihon Freezer Co., Ltd., Saitama, Japan). This study was conducted in accordance with the Institutional Animal Care and Use Committee of the Southeast Medi-Chem Institute (IRB No: SEMI-16-10).

### 3.6. Lipid Extraction from Feces and Tissues

Freeze-dried feces (0.5 g), 2 mL of pyrogallol in ethanol (50 mg/mL), and 10 mL of 8.3 M HCl were placed in a vial and vortexed for 30 s. The lipid of feces was extracted in a shaking water bath at 80 °C and 200 rpm for 1 h; the vial was taken out every 20 min and vortexed for 30 s. After extraction, 15 mL diethyl ether was added, mixed for 1 min, and centrifuged (1763× *g*, 3 min). The upper layer was mixed with 15 mL of petroleum ether and centrifuged. The supernatant was passed through a sodium sulfate column to remove moisture, and after removing the solvent with N_2_, the crude lipid content was calculated.

For lipid extraction from tissues, the liver, brain, and epididymal fat were cut into pieces and placed in a test tube, mixed with 2 mL of 0.9% saline, and homogenized using an ultrasonic processor (Sonics & Materials, Inc., Newtown, CT, USA) with 30% power for 1 min twice. The crude lipid was extracted with 12 mL of chloroform: methanol (2:1, *v*/*v*) for 30 min in a shaking water bath at 50 °C and 190 rpm, then centrifuged (441× *g*) for 5 min. The bottom layer was concentrated with N_2_. The fatty acid compositions of fecal and tissue lipids were analyzed by GC.

### 3.7. Determination of In Vivo Apparent Digestibility of Dietary Oil and the Selected Fatty Acids

Green feces obtained by feeding a chromic-oxide-supplemented diet were collected, lyophilized, and stored at −80 °C. A digestion solution was prepared with 450 mL of nitric acid, 150 mL of perchloric acid, and sodium molybdate (0.6 g). Crushed feces (250 mg) was mixed with the digestion solution (10 mL) in a 100-mL Kjeldahl flask and heated at 300 °C. The heating was continued until brownish smoke appeared from the digestion solution; at this point, the heating was stopped when the color changed to yellowish or orange. After cooling to room temperature, the digested solution was filtered, placed in a 50-mL volumetric flask, filled with distilled water, and absorbance was measured at 440 nm using a UV spectrophotometer (Optizen 2120UV, Mecasys Co., Ltd., Daejeon, Korea). After obtaining a standard curve with potassium dichromate solution, the chromic oxide content in the feces was calculated. The apparent digestibility of oil (or FA) was determined by the coefficient of digestibility, which is a measure of bioavailability expressed as the percentage of ingested oil (or FA) that was not excreted in the feces [15]. The coefficient of digestibility of oil and individual FAs was measured using the following equation:

(2) $$Coefficient\;of\;digestion\left(\%\right)=100-\left[\frac{crude\;lipid\left(fatty\;acid\right)contentin\;feces\left(\%\right)}{oil\left(fatty\;acid\right)content\;in\;diet\left(\%\right)}\times \frac{chromic\;oxide\;content\;of\;diet\left(\%\right)}{chromic\;oxide\;content\;of\;feces\left(\%\right)}\times 100\right]$$

### 3.8. Statistical Analysis

Analysis of variance (ANOVA) was performed with Statistical Analysis System 9.2 (SAS Institute Inc., Cary, NC, USA), and the statistical significance of the means was determined by Duncan’s multiple range test at *p* < 0.05.

## 4. Conclusions

The in vitro and in vivo digestion models presented consistent results on the digestibility rate of soybean, fish, and microalgal oils. The in vitro digestion rate calculated on the basis of the released FFA content was higher in soybean and fish oils than in microalgal oil, and the in vivo apparent digestibility using mice of microalgal oil (91.49%) was lower than those of soybean oil (96.50%) and fish oil (96.99%). The individual FA digestibility of diet oils was the highest for DPA, followed by EPA, DHA, palmitoleic, oleic, palmitic, and stearic acid. FA digestibility within diet groups increased with reduced chain length and increased unsaturation level. Microalgal oil contains high levels of DHA (56.8%), which has a relatively lower digestibility among PUFAs, and palmitic acid (22.4%), which has a significantly lower digestibility; consequently, its hydrolysis rate is lower than those of fish oil and soybean oil. Due to its low absorption rate in the body, the fecal lipid content is higher, leading to low apparent digestibility. The diet oils influenced the deposition of fatty acids in mouse tissues, and in particular, a significant amount of DHA was deposited in the epididymal fat, liver, and brain of mice that were fed microalgal oil. Therefore, microalgal oil shows low in vitro and in vivo digestibility, although it is incorporated into major organs, such as the brain and liver, when ingested, and could exhibit various health benefits.

## Figures and Tables

**Table 1:** Fatty acids compositions of soybean oil, fish oil, and microalgal oil.

Fatty Acid (% of Total Fatty Acids)	Soybean Oil			Fish Oil			Microalgal Oil		
	Total	*sn*-1,3	*sn*-2	Total	*sn*-1,3	*sn*-2	Total	*sn*-1,3	*sn*-2
C14:0	0.1 ± 0.0 ^(1)^	0.1 ± 0.0	- ^(2)^	9.2 ± 0.1	9.0 ± 1.1	9.8 ± 2.4	0.6 ± 0.0	0.7 ± 0.0	0.4 ± 0.0
C16:0	10.9 ± 0.0	15.8 ± 0.3	1.0 ± 0.5	18.5 ± 0.1	17.8 ± 0.7	20.0 ± 1.1	22.4 ± 0.2	27.8 ± 0.1	11.7 ± 0.8
C16:1	0.1 ± 0.0	0.1 ± 0.0	0.1 ± 0.0	13.1 ± 0.0	13.5 ± 0.5	12.3 ± 1.0	0.2 ± 0.0	0.4 ± 0.0	-
C18:0	4.5 ± 0.0	6.6 ± 0.1	0.4 ± 0.3	3.2 ± 0.0	4.4 ± 0.0	0.9 ± 0.1	1.5 ± 0.0	1.7 ± 0.1	1.1 ± 0.2
C18:1n-9c	21.7 ± 0.0	21.6 ± 0.0	22.0 ± 0.0	8.8 ± 0.0	11.6 ± 0.0	3.0 ± 0.1	0.6 ± 0.0	0.3 ± 0.0	1.1 ± 0.1
C18:1n-7c	1.3 ± 0.0	1.9 ± 0.0	0.3 ± 0.0	3.6 ± 0.1	4.0 ± 0.2	2.8 ± 0.3	0.5 ± 0.0	0.7 ± 0.0	0.3 ± 0.0
C18:2n-6c	54.6 ± 0.0	46.9 ± 0.4	70.1 ± 0.8	1.6 ± 0.0	1.8 ± 0.0	1.2 ± 0.0	1.3 ± 0.0	1.0 ± 0.0	2.0 ± 0.0
C20:0	-	-	-	0.6 ± 0.0	0.8 ± 0.0	0.2 ± 0.0	0.3 ± 0.0	0.4 ± 0.0	-
C18:3n-6	0.2 ± 0.0	0.1 ± 0.1	0.3 ± 0.1	0.5 ± 0.0	0.3 ± 0.0	0.8 ± 0.1	-	-	-
C20:1n-9	-	-	-	1.1 ± 0.0	1.4 ± 0.0	0.4 ± 0.0	-	-	-
C18:3n-3	6.6 ± 0.0	6.9 ± 0.0	5.8 ± 0.0	1.4 ± 0.0	1.6 ± 0.0	1.0 ± 0.0	0.2 ± 0.0	0.2 ± 0.0	-
C20:2	-	-	-	3.5 ± 0.0	3.2 ± 0.0	4.0 ± 0.1	0.2 ± 0.0	0.3 ± 0.0	-
C22:0	-	-	-	0.4 ± 0.3	0.5 ± 0.4	-	0.1 ± 0.0	0.2 ± 0.0	-
C20:3n-6	-	-	-	0.3 ± 0.0	0.4 ± 0.0	0.2 ± 0.1	0.2 ± 0.0	0.3 ± 0.0	-
C20:3n-3	-	-	-	0.1 ± 0.0	0.2 ± 0.0	-	0.7 ± 0.0	0.9 ± 0.0	0.3 ± 0.0
C20:4n-6 (ARA)	-	-	-	1.3 ± 0.0	1.1 ± 0.0	1.7 ± 0.1	0.2 ± 0.1	0.3 ± 0.1	-
C22:2n-6	-	-	-	1.7 ± 0.0	2.1 ± 0.0	0.7 ± 0.1	0.7 ± 0.0	0.8 ± 0.0	0.4 ± 0.0
C20:5n-3 (EPA)	-	-	-	16.2 ± 0.1	15.7 ± 0.6	17.3 ± 1.4	0.6 ± 0.0	0.7 ± 0.0	0.5 ± 0.0
C24:1n-9	-	-	-	0.3 ± 0.0	0.8 ± 0.0	-	0.1 ± 0.0	0.2 ± 0.0	-
C22:5n-6 (DPA)	-	-	-	2.6 ± 0.0	1.6 ± 0.2	4.6 ± 0.5	12.3 ± 0.0	10.9 ± 0.0	15.0 ± 0.2
C22:6n-3 (DHA)	-	-	-	10.8 ± 0.5	6.7 ± 1.9	19.0 ± 2.2	56.8 ± 0.2	51.5 ± 0.2	67.3 ± 0.9
∑SFA ^(3)^	15.5 ± 0.0	22.5 ± 0.4	1.4 ± 0.7	32.7 ± 0.3	33.5 ± 2.2	31.2 ± 3.5	25.0 ± 0.2	30.9 ± 0.2	13.2 ± 0.9
∑USFA ^(4)^	84.5 ± 0.0	77.5 ± 0.4	98.6 ± 0.7	67.3 ± 0.3	66.5 ± 2.2	68.8 ± 3.5	75.0 ± 0.2	69.1 ± 0.2	86.8 ± 0.9
∑MUFA ^(5)^	23.1 ± 0.0	23.5 ± 0.0	22.4 ± 0.0	27.5 ± 0.1	32.0 ± 0.7	18.3 ± 1.1	1.8 ± 0.0	2.1 ± 0.0	1.3 ± 0.1
∑PUFA ^(6)^	61.4 ± 0.0	54.0 ± 0.4	76.2 ± 0.7	39.8 ± 0.4	34.5 ± 2.9	50.4 ± 4.6	73.2 ± 0.2	67.0 ± 0.2	85.5 ± 1.0
∑C18-USFA	84.4 ± 0.0	77.4 ± 0.4	98.5 ± 0.7	15.9 ± 0.1	19.5 ± 0.1	8.7 ± 0.0	2.6 ± 0.0	2.2 ± 0.1	3.3 ± 0.1
∑C20-USFA	-	-	-	22.4 ± 0.1	21.8 ± 0.7	23.5 ± 1.7	1.9 ± 0.1	2.5 ± 0.1	0.7 ± 0.0
∑C22-USFA	-	-	-	15.3 ± 0.5	10.8 ± 2.1	24.3 ± 2.8	69.8 ± 0.2	63.3 ± 0.2	82.8 ± 1.0

^(1)^ Values are expressed as the mean ± standard deviation (*n* = 3); ^(2)^ Not detected; ^(3)^ total saturated fatty acids; ^(4)^ total unsaturated fatty acids; ^(5)^ total monounsaturated fatty acids; ^(6)^ total polyunsaturated fatty acids.

**Table 2:** Effect of experimental diet on body weight, tissue weight, and crude lipid content in tissues of mice.

	Normal Diet	Experimental Diet		
	NO ^(1)^	SO	FO	MO
Initial body weight (g)	26.50 ± 1.53 ^a^	28.08 ± 1.38 ^a^	27.46 ± 1.56 ^a^	27.86 ± 0.45 ^a^
Final body weight (g)	26.87 ± 1.97 ^c^	32.56 ± 3.57 ^ab^	33.24 ± 2.91 ^a^	30.33 ± 2.09 ^b^
Body weight gain (g/4 weeks)	0.37 ± 1.04 ^b^	4.45 ± 3.41 ^a^	5.55 ± 1.73 ^a^	3.28 ± 2.04 ^a^
FER (%) ^(2)^	0.48 ± 1.34 ^b^	4.88 ± 3.74 ^a^	6.54 ± 2.00 ^a^	4.23 ± 2.73 ^a^
Tissue weight (g)				
Liver	1.25 ± 0.13 ^c^	1.49 ± 0.18 ^b^	1.37 ± 0.18 ^bc^	1.85 ± 0.12 ^a^
Brain	0.47 ± 0.05	0.45 ± 0.05	0.47 ± 0.05	0.45 ± 0.05 ^NS^
Heart	0.18 ± 0.06	0.20 ± 0.08	0.18 ± 0.06	0.18 ± 0.07 ^NS^
Epididymal fat	0.30 ± 0.19 ^b^	0.70 ± 0.39 ^a^	0.82 ± 0.29 ^a^	0.63 ± 0.20 ^a^
Crude lipid content (*w*/*w* %)				
Liver	3.78 ± 0.33 ^b^	5.09 ± 0.99 ^a^	3.73 ± 0.36 ^b^	6.83 ± 2.27 ^a^
Brain	8.09 ± 0.77	8.35 ± 0.68	7.61 ± 0.11	7.46 ± 0.65 ^NS^
Epididymal fat	65.24 ± 7.76	67.55 ± 6.47	67.85 ± 5.72	67.53 ± 6.32 ^NS^

Values are expressed as the mean ± standard deviation (*n* = 10); ^(1)^ NO: soybean oil 7%, SO: soybean oil 20% FO: fish oil 20%, MO: microalgal oil 20%; ^(2)^ Feed efficiency ratio (%) = (body weight gain [g/day]/food intake [g/day]) × 100; ^a–c^ Means within the same row with different superscript letters are significantly different by Duncan’s multiple range test at *p* < 0.05; ^NS^ Means within the same row are not significantly different by Duncan’s multiple range test at *p* < 0.05.

**Table 3:** Apparent digestibility (%) of the oils and the individual fatty acids in mice.

	Normal Diet	Experimental Diets		
Coefficient of Digestibility	NO ^(1)^	SO	FO	MO
Oils	91.85 ± 0.92 ^b^	96.50 ± 1.79 ^a^	96.99 ± 0.57 ^a^	91.49 ± 1.11 ^b^
Fatty acids				
C16:0	^AB^ 88.16 ± 0.80 ^c^	^A^ 88.43 ± 0.89 ^d^	^A^ 89.22 ± 0.72 ^b^	^B^ 80.76 ± 2.41 ^c^
C16:1	- ^(2)^	-	93.83 ± 0.78 ^a^	-
C18:0	^A^ 86.49 ± 2.22 ^c^	^A^ 85.87 ± 1.79 ^e^	^B^ 76.81 ± 5.35 ^c^	-
C18:1 n-9	^A^ 92.65 ± 0.87 ^b^	^B^ 91.53 ± 0.57 ^c^	^A^ 92.74 ± 0.31 ^ab^	-
C18:2 n-6	^A^ 94.70 ± 0.76 ^ab^	^B^ 93.63 ± 0.55 ^b^	-	-
C18:3 n-3	^A^ 96.86 ± 0.59 ^a^	^A^ 96.20 ± 0.35 ^a^	-	-
C20:5(n-3, EPA)	-	-	95.65 ± 1.07 ^a^	-
C22:5(n-6, DPA)	-	-	-	99.88 ± 0.02 ^a^
C22:6(n-3, DHA)	-	-	^A^ 94.46 ± 0.83 ^a^	^A^ 93.20 ± 1.84 ^b^
Fecal crude lipids content ^(3)^	6.87 ± 0.32 ^c^	8.89 ± 0.90 ^b^	9.24 ± 0.73 ^b^	15.87 ± 2.74 ^a^

Values are expressed as the mean ± standard deviation (*n* = 10); ^(1)^ NO: soybean oil 7%, SO: soybean oil 20% FO: fish oil 20%, AO: Microalgal oil 20%; ^(2)^ not available; ^(3)^ *w*/*w*% of freeze-dried weight; ^A,B^ Means within the same row with different superscript uppercase letters are significantly different among the groups by Duncan’s multiple range test at *p* < 0.05; ^a–e^ Means within the same column with different superscript lower-case letters are significantly different among the fatty acids by Duncan’s multiple range test at *p* < 0.05.

**Table 4:** The fatty acid composition distributed in fecal lipid and epididymal fat of mice.

	**Fecal Lipid**			
**Fatty Acid ^(1)^**	**NO ^(2)^**	**SO**	**FO**	**MO**
C16:0	15.62 ± 1.03 ^c,(3)^	14.07 ± 1.08 ^c^	24.32 ± 1.63 ^b^	40.61 ± 5.07 ^a^
C16:1	0.35 ± 0.07 ^b^	0.24 ± 0.07 ^b^	9.86 ± 1.25 ^a^	0.45 ± 0.04 ^b^
C18:0	7.35 ± 1.21 ^ab^	7.09 ± 0.90 ^ab^	9.05 ± 2.09 ^a^	5.49 ± 1.40 ^b^
C18:1n-9c	19.37 ± 2.30 ^a^	20.51 ± 1.37 ^a^	7.79 ± 0.33 ^b^	1.08 ± 0.22 ^c^
C18:2n-6c	35.03 ± 5.03 ^a^	38.82 ± 3.35 ^a^	3.03 ± 0.22 ^b^	0.74 ± 0.11 ^b^
C18:3n-3	2.51 ± 0.47 ^a^	2.83 ± 0.29 ^a^	0.82 ± 0.18 ^b^	0.08 ± 0.05 ^c^
ARA(C20:4n-6)	0.76 ± 0.23 ^b^	0.41 ± 0.10 ^c^	1.54 ± 0.24 ^a^	0.46 ± 0.10 ^c^
EPA(C20:5n-3)	- ^(4)^	0.02 ± 0.05 ^b^	8.60 ± 2.12 ^a^	0.61 ± 0.14 ^b^
DPA(C22:5n-6)	-	-	1.70 ± 0.30 *	0.14 ± 0.02
DHA(C22:6n-3)	0.61 ± 0.27 ^b^	0.34 ± 0.13 ^b^	7.29 ± 1.09 ^b^	36.40 ± 9.84 ^a^
	**Epididymal fat**			
**Fatty acid**	**NO**	**SO**	**FO**	**MO**
C16:0	19.17 ± 1.44 ^c^	13.12 ± 0.46 ^d^	29.50 ± 1.14 ^b^	36.22 ± 2.67 ^a^
C16:1	4.28 ± 0.93 ^b^	2.11 ± 0.53 ^c^	9.99 ± 0.58 ^a^	3.36 ± 1.06 ^b^
C18:0	1.85 ± 1.25 ^c^	2.44 ± 0.41 ^bc^	3.76 ± 0.42 ^a^	2.95 ± 0.42 ^ab^
C18:1n-9c	34.17 ± 1.54 ^a^	31.19 ± 1.19 ^b^	21.84 ± 1.72 ^c^	17.40 ± 2.52 ^d^
C18:2n-6c	32.17 ± 0.61 ^b^	43.85 ± 1.55 ^a^	10.89 ± 0.59 ^c^	12.68 ± 1.81^c^
ARA(C20:4n-6)	0.15 ± 0.05 ^c^	0.19 ± 0.05 ^c^	0.57 ± 0.06 ^b^	1.38 ± 0.20 ^a^
EPA(C20:5n-3)	-	-	2.34 ± 0.66 *	0.78 ± 0.25
DPA(C22:5n-6)	0.01 ± 0.02 ^b^	-	0.14 ± 0.04 ^b^	2.86 ± 0.34 ^a^
DHA(C22:6n-3)	0.04 ± 0.03 ^c^	0.07 ± 0.02 ^c^	2.75 ± 0.74 ^b^	17.08 ± 1.50 ^a^

^(1)^ % of total fatty acids; ^(2)^ NO: soybean oil 7%, SO: soybean oil 20% FO: fish oil 20%, MO: Microalgal oil 20%; ^(3)^ Values are expressed as the mean ± standard deviation (*n* = 10); ^(4)^ Not detected; ^a–c^ Means within the same row with different superscript letters are significantly different according to Duncan’s multiple range test at *p* < 0.05 (*n* = 3). * Means within the same row are significantly different by Student *t*-test at *p* < 0.05.

**Table 5:** The fatty acid composition distributed in liver and brain lipid of mice.

	**Liver Lipid**			
**Fatty acid ^(1)^**	**NO ^(2)^**	**SO**	**FO**	**MO**
C16:0	20.94 ± 0.91 ^c,(3)^	19.12 ± 0.87 ^d^	26.94 ± 0.49 ^a^	24.21 ± 2.06 ^b^
C16:1	1.68 ± 0.71 ^b^	0.61 ± 0.13 ^c^	2.40 ± 0.32 ^a^	0.77 ± 0.19 ^c^
C18:0	11.49 ± 1.56 ^a^	8.74 ± 1.42 ^b^	12.75 ± 0.91 ^a^	8.46 ± 2.35 ^b^
C18:1n-9c	16.17 ± 3.42 ^a^	15.57 ± 2.50 ^a^	7.79 ± 1.35 ^b^	4.70 ± 1.88 ^b^
C18:2n-6c	21.01 ± 1.51 ^b^	33.24 ± 2.01 ^a^	7.14 ± 0.89 ^c^	4.49 ± 2.14 ^d^
C18:3n-3	0.25 ± 0.05 ^b^	0.79 ± 0.11 ^a^	0.33 ± 0.16 ^b^	0.11 ± 0.05 ^c^
ARA(C20:4n-6)	14.44 ± 2.60 ^a^	10.07 ± 1.64 ^b^	8.52 ± 0.76 ^bc^	6.72 ± 1.28 ^c^
EPA(C20:5n-3)	0.12 ± 0.04 ^c^	0.12 ± 0.01 ^c^	4.21 ± 0.74 ^a^	2.47 ± 0.56 ^b^
DPA(C22:5n-6)	0.54 ± 0.16 ^b^	0.14 ± 0.04 ^c^	0.24 ± 0.01 ^c^	4.19 ± 0.38 ^a^
DHA(C22:6n-3)	7.22 ± 0.44 ^c^	5.69 ± 1.20 ^c^	24.01 ± 1.55 ^b^	40.33 ± 1.98 ^a^
	**Brain Lipid**			
**Fatty acid**	**NO**	**SO**	**FO**	**MO**
C16:0	22.42 ± 0.34 ^a^	22.18 ± 0.19 ^ab^	22.08 ± 0.12 ^b^	22.39 ± 0.15 ^a^
C18:0	20.66 ± 0.26 ^b^	20.59 ± 0.25 ^b^	20.59 ± 0.29 ^b^	21.54 ± 0.31 ^a^
C18:1n-9c	18.21 ± 0.45 ^b^	18.44 ± 0.54 ^b^	19.60 ± 0.32 ^a^	16.58 ± 0.57 ^c^
C18:2n-6c	0.93 ± 0.07 ^b^	1.72 ± 0.55 ^a^	0.40 ± 0.06 ^c^	0.29 ± 0.04 ^c^
ARA(C20:4n-6)	10.29 ± 0.20 ^a^	10.04 ± 0.26 ^a^	7.98 ± 0.16 ^b^	7.78 ± 0.31 ^b^
EPA(C20:5n-3)	- ^(4)^	-	0.36 ± 0.02 *	0.23 ± 0.02
DPA(C22:5n-6)	0.41 ± 0.03 ^b^	0.30 ± 0.02 ^c^	0.30 ± 0.02 ^c^	1.78 ± 0.06 ^a^
DHA(C22:6n-3)	15.65 ± 0.31 ^c^	15.36 ± 0.49 ^c^	17.76 ± 0.28 ^b^	20.43 ± 0.48 ^a^

^(1)^ % of total fatty acids; ^(2)^ NO: soybean oil 7%, SO: soybean oil 20% FO: fish oil 20%, MO: Microalgal oil 20%; ^(3)^ Values are expressed as the mean ± standard deviation (*n* = 10); ^(4)^ Not detected; ^a–c^ Means within the same row with different superscript letters are significantly different by Duncan’s multiple range test at *p* < 0.05 (*n* = 3). * Means within the same row are significantly different by Student *t*-test at *p* < 0.05.

**Table 6:** The composition of the animal diet.

Ingredient		Normal Diet (Oil 7%)	Experimental Diet (Oil 20%)		
		NO ^(1)^	SO	FO	MO
Casein		20.00	20.00	20.00	20.00
Corn Starch		39.75	26.75	26.75	26.75
Maltodextrin		13.20	13.20	13.20	13.20
Sucrose		10.00	10.00	10.00	10.00
Oil	Soybean oil	7.00	20.00	-	-
	Fish oil	-	-	20.00	-
	Microalgal oil	-	-	-	20.00
Cellulose		5.00	5.00	5.00	5.00
AIN-93G Mineral mixture ^(2)^		3.50	3.5	3.5	3.5
AIN-93G Vitamin mixture ^(3)^		1.00	1.00	1.00	1.00
L-cysteine		0.30	0.30	0.30	0.30
Choline Bitartrate		0.245	0.25	0.25	0.25
t-Butylhydroquinone		0.0014	0.0014	0.0014	0.0014
Total		100.00	100.00	100.00	100.00

^(1)^ NO: soybean oil 7%, SO: soybean oil 20% FO: fish oil 20%, MO: microalgal oil 20%; ^(2)^ calcium 0.51%, phosphorus 0.32%, potassium 0.36%, magnesium 0.05%, sodium 0.31%, chloride 0.22%, fluorine 1.0 ppm, iron 40 ppm, zine 35 ppm, manganese 11 ppm, popper 6.0 ppm, iodine 0.21 ppm, chromium 0.14 ppm, selenium 0.24 ppm; ^(3)^ vitamin A 4.0 IU/g, vitamin D 1.0 IU/g, vitamin E 81.6 IU/kg, vitamin K 0.75 ppm, thiamin 4.8 ppm, riboflavin 6.7 ppm, niacin 30 ppm, pantothenic acid 16 ppm, folic acid 2.1 ppm, pyridoxine 5.8 ppm, biotin 0.2 ppm, vitamin B-12 28 mcg/kg, choline chloride 1250 ppm.
